# Dibenzyl ferrocene-1,1′-dicarboxyl­ate

**DOI:** 10.1107/S1600536811016588

**Published:** 2011-05-14

**Authors:** Brian O. Patrick, Chris Rock, Alaa S. Abd-El-Aziz

**Affiliations:** a Department of Chemistry, University of British Columbia, 2036 Main Mall, Vancouver, BC, Canada V6T 1Z1; b Department of Chemistry, University of British Columbia, Okanagan Campus, 3333 University Way, Kelowna, BC, Canada V1V 1V7

## Abstract

In the title compound, [Fe(C_13_H_11_O_2_)_2_], there are markedly different orientations of the two phenyl­meth­oxy­carbonyl substituents [O—C—C—C torsion angles = 84.5 (3) and 139.6 (2)°]. These orientations are mediated by a number of inter­molecular C—H⋯O inter­actions, which result in a one-dimensional hydrogen-bonded network of mol­ecules.

## Related literature

For properties of ferrocene-incorporated compounds, see: Abd-El-Aziz *et al.* (2007 ▶). For the crystal structure of a ferroecene ester, see: Hur *et al.* (2010 ▶). For the crystallization of monoacetylferrocene, see: Khrustalev *et al.* (2006 ▶).
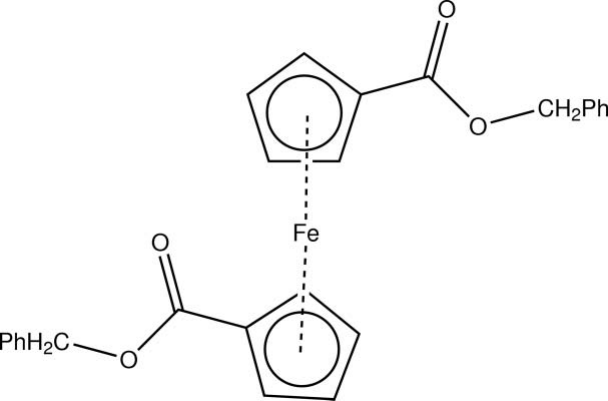


## Experimental

### 

#### Crystal data

[Fe(C_13_H_11_O_2_)_2_]
*M* *_r_* = 454.29
Orthorhombic, 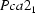

*a* = 13.1120 (14) Å
*b* = 5.9366 (6) Å
*c* = 25.538 (3) Å
*V* = 1987.9 (4) Å^3^
*Z* = 4
Mo *K*α radiation
μ = 0.79 mm^−1^
*T* = 100 K
0.15 × 0.05 × 0.05 mm

#### Data collection

Bruker X8 APEXII diffractometer
Absorption correction: multi-scan (*SADABS*; Bruker, 2008 ▶) *T*_min_ = 0.830, *T*_max_ = 0.961
16738 measured reflections
4726 independent reflections
3820 reflections with *I* > 2σ(*I*)
*R*_int_ = 0.050

#### Refinement

*R*[*F* ^2^ > 2σ(*F* ^2^)] = 0.035
*wR*(*F* ^2^) = 0.070
*S* = 1.02
4726 reflections
280 parameters
1 restraint
H-atom parameters constrained
Δρ_max_ = 0.31 e Å^−3^
Δρ_min_ = −0.33 e Å^−3^
Absolute structure: Flack (1983 ▶), 2280 Friedel pairs
Flack parameter: 0.016 (14)

### 

Data collection: *APEX2* (Bruker, 2008 ▶); cell refinement: *SAINT* (Bruker, 2008 ▶); data reduction: *SAINT*; program(s) used to solve structure: *SIR97* (Altomare *et al.*, 1999 ▶); program(s) used to refine structure: *SHELXL97* (Sheldrick, 2008 ▶); molecular graphics: *ORTEP-3 for Windows* (Farrugia, 1997 ▶); software used to prepare material for publication: *WinGX* (Farrugia, 1999 ▶).

## Supplementary Material

Crystal structure: contains datablocks I, global. DOI: 10.1107/S1600536811016588/pv2401sup1.cif

Structure factors: contains datablocks I. DOI: 10.1107/S1600536811016588/pv2401Isup2.hkl

Additional supplementary materials:  crystallographic information; 3D view; checkCIF report

## Figures and Tables

**Table 1:** Hydrogen-bond geometry (Å, °)

*D*—H⋯*A*	*D*—H	H⋯*A*	*D*⋯*A*	*D*—H⋯*A*
C1—H1⋯O2^i^	0.95	2.46	3.368 (3)	160
C7—H7*B*⋯O1^ii^	0.99	2.44	3.350 (3)	152
C9—H9⋯O1^ii^	0.95	2.59	3.374 (3)	141
C20—H20*B*⋯O3^ii^	0.99	2.54	3.421 (3)	148
C22—H22⋯O3^ii^	0.95	2.51	3.233 (3)	133

Symmetry codes: (i) 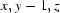; (ii) 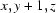.

**Fig. 1.**
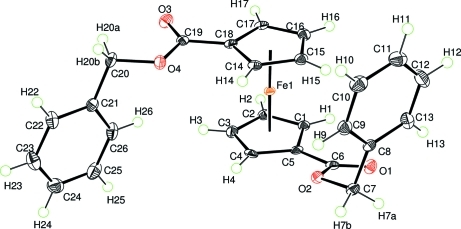
The molecular structure of the title compound, with atomic labels and 50% probability displacement ellipsoids.

**Fig. 2.**
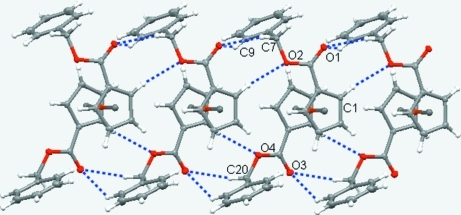
One-dimensional C—H···O hydrogen bonded network formed by the title compound.

## supplementary crystallographic information

### Comment

Ferrocene has well defined redox properties and has successfully been incorporated into larger compounds in order to take advantage of these properties (Abd-El-Aziz *et al.*, 2007). The monoester of the title compound was reported as a classic example of a ferroecene ester (Hur *et al.*, 2010).

The stucture of the title compound is presented in Fig. 1. While the molecule posesses internal inversion symmetry, this symmetry is broken in the solid state. The two benzyl cyclopentadienylcarboxylate groups stack almost perfectly eclipsed. However, there is a significant difference between the orientation of the two benzyl substituents, as denoted by the O2—C7—C8—C9 and O4—C20—C21—C22 torsion angles [84.5 (3) and 139.6 (2)°, respectively]. Of the two torsion angles described above, the former resembles most closely that of the monoester, with a torsion angle of 82.4° (Hur *et al.*, 2010). These different orientations are mediated by a variety of intermolecular C—H···O interactions. These interactions ultimately form a one-dimensional hydrogen-bonded network of molecules; details have been given in Table 1 and Figure 2.

### Experimental

The title compound was formed during attempts to incorporate ferrocene along with free arenes into larger dendrimers and polymers. 1,1'-Ddicarboxylic acid ferrocene (4 mmol), benzyl alcohol (4 mmol), *N,N'*-dicyclohexylcarbodiimide(DCC) (4.4 mmol) and dimethylaminopyride (DMAP) (4.4 mmol) were combined in 50 mL of dimethylformamide (DMF) and stirred under an atmosphere of nitrogen for 15 h. The mixture was then cooled to 269 K for 30 minutes before gravity filtration. The filtrate was washed with 1.2 *M* HCl and deionized water consecutively. The solution was then dried using MgSO_4_ and the solvent removed *in vacuo*. The di-substituted form was separated from the mono-substituted (*i.e.*, monoester) form using a silica gel column, using ethyl acetate as the eluent. Red crystals of the product were grown using ethyl acetate.

### Refinement

All hydrogen atoms were placed in calculated positions, riding on C atoms with *U*_iso_(H) = 1.2*U*_eq_(C). The Cp and phenyl H atoms were placed at C—H = 0.95 Å and methylene H atoms at C—H = 0.99 Å. The absolute stereochemistry was determined on the basis of the refined Flack *x*-parameter, with *x* = 0.02 (1) (Flack, 1983) using 2280 Friedel pairs of reflections which were not merged.

### Crystal data

**Table:**

[Fe(C_13_H_11_O_2_)_2_]	*F*(000) = 944
*M**_r_* = 454.29	*D*_x_ = 1.518 Mg m^−^^3^
Orthorhombic, *P**c**a*2_1_	Mo *K*α radiation, λ = 0.71073 Å
Hall symbol: P 2c -2a2	Cell parameters from 3257 reflections
*a* = 13.1120 (14) Å	θ = 3.2–26.1°
*b* = 5.9366 (6) Å	µ = 0.79 mm^−^^1^
*c* = 25.538 (3) Å	*T* = 100 K
*V* = 1987.9 (4) Å^3^	Irregular, orange
*Z* = 4	0.15 × 0.05 × 0.05 mm

### Data collection

**Table:**

Bruker X8 APEXII diffractometer	4726 independent reflections
Radiation source: fine-focus sealed tube	3820 reflections with *I* > 2σ(*I*)
graphite	*R*_int_ = 0.050
area detector scans	θ_max_ = 28.0°, θ_min_ = 1.6°
Absorption correction: multi-scan (*SADABS*; Bruker, 2008)	*h* = −14→17
*T*_min_ = 0.830, *T*_max_ = 0.961	*k* = −7→7
16738 measured reflections	*l* = −33→33

### Refinement

**Table:**

Refinement on *F*^2^	Secondary atom site location: difference Fourier map
Least-squares matrix: full	Hydrogen site location: inferred from neighbouring sites
*R*[*F*^2^ > 2σ(*F*^2^)] = 0.035	H-atom parameters constrained
*wR*(*F*^2^) = 0.070	*w* = 1/[σ^2^(*F*_o_^2^) + (0.0277*P*)^2^ + 0.1317*P*] where *P* = (*F*_o_^2^ + 2*F*_c_^2^)/3
*S* = 1.02	(Δ/σ)_max_ = 0.002
4726 reflections	Δρ_max_ = 0.31 e Å^−^^3^
280 parameters	Δρ_min_ = −0.33 e Å^−^^3^
1 restraint	Absolute structure: Flack (1983), 2280 Friedel pairs
Primary atom site location: structure-invariant direct methods	Flack parameter: 0.016 (14)

### Special details

**Table:**

Experimental. Data were scaled in point group 222 in order to keep all Friedel pairs unmerged. In total 97% of all Friedel pairs were measured.
Geometry. All e.s.d.'s (except the e.s.d. in the dihedral angle between two l.s. planes) are estimated using the full covariance matrix. The cell e.s.d.'s are taken into account individually in the estimation of e.s.d.'s in distances, angles and torsion angles; correlations between e.s.d.'s in cell parameters are only used when they are defined by crystal symmetry. An approximate (isotropic) treatment of cell e.s.d.'s is used for estimating e.s.d.'s involving l.s. planes.
Refinement. Refinement of *F*^2^ against ALL reflections. The weighted *R*-factor *wR* and goodness of fit *S* are based on *F*^2^, conventional *R*-factors *R* are based on *F*, with *F* set to zero for negative *F*^2^. The threshold expression of *F*^2^ > σ(*F*^2^) is used only for calculating *R*-factors(gt) *etc*. and is not relevant to the choice of reflections for refinement. *R*-factors based on *F*^2^ are statistically about twice as large as those based on *F*, and *R*- factors based on ALL data will be even larger.

### Fractional atomic coordinates and isotropic or equivalent isotropic displacement parameters (Å^2^)

**Table:**

	*x*	*y*	*z*	*U*_iso_*/*U*_eq_	
C1	0.01829 (18)	0.0437 (4)	0.19218 (9)	0.0126 (5)	
H1	0.0185	−0.0791	0.2160	0.015*	
C2	0.0484 (2)	0.0360 (4)	0.13853 (10)	0.0147 (5)	
H2	0.0730	−0.0928	0.1204	0.018*	
C3	0.03515 (19)	0.2546 (5)	0.11696 (10)	0.0168 (5)	
H3	0.0482	0.2964	0.0817	0.020*	
C4	−0.0009 (2)	0.3998 (5)	0.15699 (9)	0.0137 (6)	
H4	−0.0149	0.5560	0.1534	0.016*	
C5	−0.01225 (18)	0.2695 (4)	0.20357 (10)	0.0130 (5)	
C6	−0.04033 (19)	0.3471 (4)	0.25657 (10)	0.0132 (5)	
C7	−0.0510 (2)	0.6662 (4)	0.31290 (10)	0.0148 (5)	
H7A	−0.0999	0.5714	0.3326	0.018*	
H7B	−0.0796	0.8201	0.3103	0.018*	
C8	0.0496 (2)	0.6733 (4)	0.34143 (9)	0.0145 (6)	
C9	0.1152 (2)	0.8552 (5)	0.33434 (10)	0.0175 (6)	
H9	0.0948	0.9771	0.3126	0.021*	
C10	0.2096 (2)	0.8607 (5)	0.35846 (11)	0.0229 (7)	
H10	0.2536	0.9858	0.3533	0.027*	
C11	0.2400 (2)	0.6838 (5)	0.39020 (11)	0.0250 (7)	
H11	0.3050	0.6866	0.4066	0.030*	
C12	0.1750 (2)	0.5026 (5)	0.39792 (11)	0.0259 (7)	
H12	0.1952	0.3819	0.4200	0.031*	
C13	0.0805 (2)	0.4967 (5)	0.37356 (10)	0.0197 (6)	
H13	0.0366	0.3715	0.3788	0.024*	
C14	0.22644 (18)	0.5344 (4)	0.19765 (9)	0.0130 (5)	
H14	0.2071	0.6886	0.1971	0.016*	
C15	0.21971 (19)	0.3869 (4)	0.24127 (10)	0.0149 (5)	
H15	0.1952	0.4252	0.2751	0.018*	
C16	0.2561 (2)	0.1719 (4)	0.22540 (10)	0.0156 (5)	
H16	0.2601	0.0417	0.2469	0.019*	
C17	0.28541 (17)	0.1843 (4)	0.17215 (11)	0.0137 (5)	
H17	0.3122	0.0645	0.1517	0.016*	
C18	0.26740 (19)	0.4098 (4)	0.15472 (9)	0.0126 (5)	
C19	0.29004 (18)	0.4912 (4)	0.10128 (10)	0.0128 (5)	
C20	0.2863 (2)	0.8194 (5)	0.04625 (10)	0.0188 (6)	
H20A	0.3273	0.7167	0.0241	0.023*	
H20B	0.3269	0.9572	0.0527	0.023*	
C21	0.1895 (2)	0.8802 (5)	0.01829 (10)	0.0146 (6)	
C22	0.1823 (2)	1.0860 (5)	−0.00775 (10)	0.0204 (6)	
H22	0.2376	1.1893	−0.0064	0.025*	
C23	0.0947 (2)	1.1407 (5)	−0.03571 (11)	0.0250 (6)	
H23	0.0912	1.2792	−0.0543	0.030*	
C24	0.0130 (2)	0.9952 (5)	−0.03662 (11)	0.0264 (7)	
H24	−0.0472	1.0347	−0.0551	0.032*	
C25	0.0187 (2)	0.7918 (5)	−0.01058 (10)	0.0257 (7)	
H25	−0.0375	0.6910	−0.0113	0.031*	
C26	0.1066 (2)	0.7348 (5)	0.01658 (11)	0.0216 (6)	
H26	0.1101	0.5943	0.0343	0.026*	
O1	−0.06304 (14)	0.2228 (3)	0.29215 (7)	0.0176 (4)	
O2	−0.03591 (13)	0.5731 (3)	0.26035 (6)	0.0134 (4)	
O3	0.32897 (14)	0.3805 (3)	0.06757 (7)	0.0185 (4)	
O4	0.26322 (14)	0.7100 (3)	0.09598 (7)	0.0167 (4)	
Fe1	0.13475 (2)	0.26824 (5)	0.179289 (17)	0.01072 (8)	

### Atomic displacement parameters (Å^2^)

**Table:**

	*U*^11^	*U*^22^	*U*^33^	*U*^12^	*U*^13^	*U*^23^
C1	0.0091 (12)	0.0132 (12)	0.0156 (14)	−0.0014 (10)	−0.0005 (9)	0.0005 (9)
C2	0.0097 (12)	0.0179 (13)	0.0164 (12)	−0.0032 (11)	−0.0006 (10)	−0.0038 (11)
C3	0.0120 (13)	0.0238 (15)	0.0146 (12)	−0.0039 (11)	−0.0016 (10)	0.0022 (11)
C4	0.0067 (12)	0.0147 (14)	0.0197 (13)	0.0005 (11)	−0.0050 (10)	0.0016 (11)
C5	0.0048 (12)	0.0162 (13)	0.0181 (11)	−0.0022 (11)	0.0001 (9)	0.0017 (11)
C6	0.0068 (13)	0.0137 (13)	0.0191 (13)	0.0003 (10)	−0.0008 (10)	0.0005 (10)
C7	0.0137 (14)	0.0154 (13)	0.0155 (12)	0.0018 (11)	0.0033 (11)	−0.0017 (10)
C8	0.0134 (14)	0.0171 (14)	0.0129 (12)	0.0038 (11)	0.0035 (10)	−0.0016 (10)
C9	0.0182 (16)	0.0181 (15)	0.0161 (13)	0.0011 (12)	0.0020 (11)	−0.0007 (11)
C10	0.0183 (16)	0.0288 (17)	0.0214 (14)	−0.0038 (13)	−0.0006 (12)	−0.0048 (13)
C11	0.0182 (16)	0.0400 (18)	0.0168 (13)	0.0038 (13)	−0.0043 (11)	−0.0096 (12)
C12	0.0310 (18)	0.0289 (17)	0.0178 (14)	0.0087 (14)	−0.0054 (12)	0.0031 (12)
C13	0.0241 (16)	0.0191 (15)	0.0160 (13)	0.0009 (12)	−0.0006 (11)	0.0022 (11)
C14	0.0074 (12)	0.0140 (12)	0.0175 (12)	−0.0022 (10)	−0.0033 (9)	−0.0019 (10)
C15	0.0112 (13)	0.0197 (14)	0.0140 (12)	−0.0008 (11)	−0.0016 (10)	−0.0021 (10)
C16	0.0125 (13)	0.0168 (13)	0.0175 (13)	−0.0035 (11)	−0.0039 (11)	0.0024 (10)
C17	0.0068 (11)	0.0141 (12)	0.0202 (15)	−0.0002 (8)	0.0001 (11)	−0.0016 (11)
C18	0.0065 (12)	0.0145 (13)	0.0170 (12)	−0.0012 (11)	−0.0001 (10)	−0.0005 (10)
C19	0.0070 (12)	0.0140 (13)	0.0172 (12)	−0.0039 (11)	−0.0028 (10)	0.0020 (10)
C20	0.0187 (15)	0.0198 (15)	0.0181 (13)	−0.0028 (12)	0.0053 (11)	0.0057 (11)
C21	0.0163 (15)	0.0163 (14)	0.0112 (12)	−0.0005 (12)	0.0027 (11)	−0.0027 (11)
C22	0.0270 (16)	0.0180 (14)	0.0163 (13)	−0.0035 (12)	−0.0021 (12)	0.0033 (11)
C23	0.0338 (18)	0.0241 (15)	0.0171 (14)	0.0017 (14)	−0.0042 (13)	0.0030 (11)
C24	0.0221 (16)	0.0402 (18)	0.0170 (13)	0.0047 (14)	−0.0032 (12)	−0.0011 (13)
C25	0.0230 (15)	0.0371 (18)	0.0169 (14)	−0.0096 (14)	0.0016 (12)	−0.0005 (13)
C26	0.0276 (16)	0.0201 (15)	0.0171 (13)	−0.0064 (13)	0.0019 (11)	0.0046 (12)
O1	0.0211 (10)	0.0130 (9)	0.0187 (9)	0.0000 (8)	0.0052 (7)	0.0016 (8)
O2	0.0140 (9)	0.0107 (9)	0.0155 (9)	0.0006 (7)	0.0019 (7)	0.0006 (7)
O3	0.0195 (11)	0.0174 (10)	0.0185 (9)	0.0003 (8)	0.0044 (8)	−0.0026 (8)
O4	0.0194 (10)	0.0157 (10)	0.0150 (9)	0.0029 (8)	0.0036 (7)	0.0024 (7)
Fe1	0.00747 (14)	0.01212 (15)	0.01259 (13)	−0.00068 (14)	−0.00029 (19)	0.0001 (2)

### Geometric parameters (Å, °)

**Table:**

C1—C2	1.426 (4)	C14—C15	1.419 (3)
C1—C5	1.429 (3)	C14—C18	1.427 (3)
C1—Fe1	2.053 (2)	C14—Fe1	2.040 (2)
C1—H1	0.9500	C14—H14	0.9500
C2—C3	1.420 (4)	C15—C16	1.422 (4)
C2—Fe1	2.066 (2)	C15—Fe1	2.060 (2)
C2—H2	0.9500	C15—H15	0.9500
C3—C4	1.418 (4)	C16—C17	1.415 (4)
C3—Fe1	2.061 (2)	C16—Fe1	2.061 (3)
C3—H3	0.9500	C16—H16	0.9500
C4—C5	1.427 (3)	C17—C18	1.431 (3)
C4—Fe1	2.024 (3)	C17—Fe1	2.046 (2)
C4—H4	0.9500	C17—H17	0.9500
C5—C6	1.476 (3)	C18—C19	1.478 (3)
C5—Fe1	2.025 (2)	C18—Fe1	2.031 (2)
C6—O1	1.208 (3)	C19—O3	1.197 (3)
C6—O2	1.346 (3)	C19—O4	1.353 (3)
C7—O2	1.465 (3)	C20—O4	1.458 (3)
C7—C8	1.508 (4)	C20—C21	1.501 (4)
C7—H7A	0.9900	C20—H20A	0.9900
C7—H7B	0.9900	C20—H20B	0.9900
C8—C13	1.391 (3)	C21—C26	1.389 (4)
C8—C9	1.392 (4)	C21—C22	1.394 (4)
C9—C10	1.383 (4)	C22—C23	1.391 (4)
C9—H9	0.9500	C22—H22	0.9500
C10—C11	1.385 (4)	C23—C24	1.376 (4)
C10—H10	0.9500	C23—H23	0.9500
C11—C12	1.386 (4)	C24—C25	1.380 (4)
C11—H11	0.9500	C24—H24	0.9500
C12—C13	1.387 (4)	C25—C26	1.386 (4)
C12—H12	0.9500	C25—H25	0.9500
C13—H13	0.9500	C26—H26	0.9500
			
C2—C1—C5	107.7 (2)	C14—C18—C17	107.9 (2)
C2—C1—Fe1	70.20 (14)	C14—C18—C19	128.0 (2)
C5—C1—Fe1	68.42 (14)	C17—C18—C19	124.1 (2)
C2—C1—H1	126.2	C14—C18—Fe1	69.81 (14)
C5—C1—H1	126.2	C17—C18—Fe1	69.99 (13)
Fe1—C1—H1	126.8	C19—C18—Fe1	126.34 (18)
C3—C2—C1	108.0 (2)	O3—C19—O4	124.5 (2)
C3—C2—Fe1	69.67 (14)	O3—C19—C18	124.8 (2)
C1—C2—Fe1	69.28 (15)	O4—C19—C18	110.7 (2)
C3—C2—H2	126.0	O4—C20—C21	110.2 (2)
C1—C2—H2	126.0	O4—C20—H20A	109.6
Fe1—C2—H2	126.6	C21—C20—H20A	109.6
C4—C3—C2	108.5 (2)	O4—C20—H20B	109.6
C4—C3—Fe1	68.30 (14)	C21—C20—H20B	109.6
C2—C3—Fe1	70.06 (14)	H20A—C20—H20B	108.1
C4—C3—H3	125.8	C26—C21—C22	118.5 (3)
C2—C3—H3	125.8	C26—C21—C20	121.9 (2)
Fe1—C3—H3	127.4	C22—C21—C20	119.6 (2)
C3—C4—C5	107.8 (2)	C23—C22—C21	120.3 (3)
C3—C4—Fe1	71.08 (15)	C23—C22—H22	119.8
C5—C4—Fe1	69.40 (14)	C21—C22—H22	119.8
C3—C4—H4	126.1	C24—C23—C22	120.3 (3)
C5—C4—H4	126.1	C24—C23—H23	119.8
Fe1—C4—H4	125.0	C22—C23—H23	119.8
C4—C5—C1	108.0 (2)	C23—C24—C25	119.9 (3)
C4—C5—C6	128.4 (2)	C23—C24—H24	120.0
C1—C5—C6	123.3 (2)	C25—C24—H24	120.0
C4—C5—Fe1	69.33 (14)	C24—C25—C26	120.0 (3)
C1—C5—Fe1	70.57 (14)	C24—C25—H25	120.0
C6—C5—Fe1	121.29 (17)	C26—C25—H25	120.0
O1—C6—O2	124.4 (2)	C25—C26—C21	120.9 (3)
O1—C6—C5	124.1 (2)	C25—C26—H26	119.5
O2—C6—C5	111.5 (2)	C21—C26—H26	119.5
O2—C7—C8	109.6 (2)	C6—O2—C7	115.83 (19)
O2—C7—H7A	109.8	C19—O4—C20	117.4 (2)
C8—C7—H7A	109.8	C4—Fe1—C5	41.27 (10)
O2—C7—H7B	109.8	C4—Fe1—C18	120.38 (10)
C8—C7—H7B	109.8	C5—Fe1—C18	155.26 (10)
H7A—C7—H7B	108.2	C4—Fe1—C14	106.47 (10)
C13—C8—C9	118.8 (3)	C5—Fe1—C14	119.19 (10)
C13—C8—C7	121.2 (2)	C18—Fe1—C14	41.05 (9)
C9—C8—C7	120.0 (2)	C4—Fe1—C17	156.55 (10)
C10—C9—C8	120.9 (3)	C5—Fe1—C17	161.36 (10)
C10—C9—H9	119.6	C18—Fe1—C17	41.09 (10)
C8—C9—H9	119.6	C14—Fe1—C17	68.91 (10)
C9—C10—C11	120.0 (3)	C4—Fe1—C1	69.04 (10)
C9—C10—H10	120.0	C5—Fe1—C1	41.01 (10)
C11—C10—H10	120.0	C18—Fe1—C1	162.74 (10)
C10—C11—C12	119.7 (3)	C14—Fe1—C1	154.70 (10)
C10—C11—H11	120.2	C17—Fe1—C1	125.05 (10)
C12—C11—H11	120.2	C4—Fe1—C15	124.01 (11)
C11—C12—C13	120.3 (3)	C5—Fe1—C15	106.15 (10)
C11—C12—H12	119.8	C18—Fe1—C15	68.45 (10)
C13—C12—H12	119.8	C14—Fe1—C15	40.51 (10)
C12—C13—C8	120.3 (3)	C17—Fe1—C15	68.26 (10)
C12—C13—H13	119.8	C1—Fe1—C15	120.07 (10)
C8—C13—H13	119.8	C4—Fe1—C16	161.16 (10)
C15—C14—C18	107.9 (2)	C5—Fe1—C16	124.14 (10)
C15—C14—Fe1	70.49 (14)	C18—Fe1—C16	68.29 (10)
C18—C14—Fe1	69.14 (14)	C14—Fe1—C16	68.19 (10)
C15—C14—H14	126.1	C17—Fe1—C16	40.31 (10)
C18—C14—H14	126.1	C1—Fe1—C16	107.61 (10)
Fe1—C14—H14	125.9	C15—Fe1—C16	40.38 (10)
C14—C15—C16	108.0 (2)	C4—Fe1—C3	40.62 (10)
C14—C15—Fe1	69.00 (14)	C5—Fe1—C3	68.50 (10)
C16—C15—Fe1	69.85 (14)	C18—Fe1—C3	108.69 (10)
C14—C15—H15	126.0	C14—Fe1—C3	125.56 (10)
C16—C15—H15	126.0	C17—Fe1—C3	122.25 (10)
Fe1—C15—H15	126.7	C1—Fe1—C3	68.09 (10)
C17—C16—C15	108.6 (2)	C15—Fe1—C3	161.78 (11)
C17—C16—Fe1	69.26 (14)	C16—Fe1—C3	156.92 (11)
C15—C16—Fe1	69.77 (15)	C4—Fe1—C2	68.54 (11)
C17—C16—H16	125.7	C5—Fe1—C2	68.59 (10)
C15—C16—H16	125.7	C18—Fe1—C2	126.16 (10)
Fe1—C16—H16	126.8	C14—Fe1—C2	162.92 (10)
C16—C17—C18	107.6 (2)	C17—Fe1—C2	108.77 (10)
C16—C17—Fe1	70.42 (14)	C1—Fe1—C2	40.52 (10)
C18—C17—Fe1	68.92 (13)	C15—Fe1—C2	155.79 (10)
C16—C17—H17	126.2	C16—Fe1—C2	121.74 (10)
C18—C17—H17	126.2	C3—Fe1—C2	40.27 (10)
Fe1—C17—H17	126.0		
			
C5—C1—C2—C3	−0.8 (3)	C14—C18—Fe1—C1	−160.7 (3)
Fe1—C1—C2—C3	−59.10 (17)	C17—C18—Fe1—C1	−41.8 (4)
C5—C1—C2—Fe1	58.34 (17)	C19—C18—Fe1—C1	76.4 (4)
C1—C2—C3—C4	1.3 (3)	C14—C18—Fe1—C15	−37.69 (14)
Fe1—C2—C3—C4	−57.60 (18)	C17—C18—Fe1—C15	81.20 (15)
C1—C2—C3—Fe1	58.85 (17)	C19—C18—Fe1—C15	−160.6 (3)
C2—C3—C4—C5	−1.3 (3)	C14—C18—Fe1—C16	−81.28 (15)
Fe1—C3—C4—C5	−59.93 (17)	C17—C18—Fe1—C16	37.61 (15)
C2—C3—C4—Fe1	58.68 (18)	C19—C18—Fe1—C16	155.8 (3)
C3—C4—C5—C1	0.8 (3)	C14—C18—Fe1—C3	123.13 (15)
Fe1—C4—C5—C1	−60.23 (17)	C17—C18—Fe1—C3	−117.98 (15)
C3—C4—C5—C6	175.2 (2)	C19—C18—Fe1—C3	0.2 (3)
Fe1—C4—C5—C6	114.2 (3)	C14—C18—Fe1—C2	164.50 (14)
C3—C4—C5—Fe1	61.00 (18)	C17—C18—Fe1—C2	−76.62 (18)
C2—C1—C5—C4	0.0 (3)	C19—C18—Fe1—C2	41.5 (3)
Fe1—C1—C5—C4	59.45 (16)	C15—C14—Fe1—C4	123.50 (15)
C2—C1—C5—C6	−174.7 (2)	C18—C14—Fe1—C4	−117.61 (15)
Fe1—C1—C5—C6	−115.3 (2)	C15—C14—Fe1—C5	80.60 (17)
C2—C1—C5—Fe1	−59.45 (17)	C18—C14—Fe1—C5	−160.51 (14)
C4—C5—C6—O1	168.6 (2)	C15—C14—Fe1—C18	−118.9 (2)
C1—C5—C6—O1	−17.8 (4)	C15—C14—Fe1—C17	−80.81 (16)
Fe1—C5—C6—O1	−104.0 (3)	C18—C14—Fe1—C17	38.08 (14)
C4—C5—C6—O2	−12.4 (4)	C15—C14—Fe1—C1	47.8 (3)
C1—C5—C6—O2	161.2 (2)	C18—C14—Fe1—C1	166.7 (2)
Fe1—C5—C6—O2	75.0 (2)	C18—C14—Fe1—C15	118.9 (2)
O2—C7—C8—C13	−93.1 (3)	C15—C14—Fe1—C16	−37.36 (15)
O2—C7—C8—C9	84.5 (3)	C18—C14—Fe1—C16	81.54 (15)
C13—C8—C9—C10	0.3 (4)	C15—C14—Fe1—C3	163.89 (15)
C7—C8—C9—C10	−177.3 (2)	C18—C14—Fe1—C3	−77.21 (17)
C8—C9—C10—C11	−0.1 (4)	C15—C14—Fe1—C2	−166.2 (3)
C9—C10—C11—C12	−0.5 (4)	C18—C14—Fe1—C2	−47.3 (4)
C10—C11—C12—C13	0.8 (4)	C16—C17—Fe1—C4	163.5 (2)
C11—C12—C13—C8	−0.5 (4)	C18—C17—Fe1—C4	44.7 (3)
C9—C8—C13—C12	−0.1 (4)	C16—C17—Fe1—C5	−38.7 (4)
C7—C8—C13—C12	177.6 (2)	C18—C17—Fe1—C5	−157.5 (3)
C18—C14—C15—C16	−0.1 (3)	C16—C17—Fe1—C18	118.8 (2)
Fe1—C14—C15—C16	59.15 (18)	C16—C17—Fe1—C14	80.74 (16)
C18—C14—C15—Fe1	−59.27 (16)	C18—C17—Fe1—C14	−38.04 (14)
C14—C15—C16—C17	−0.1 (3)	C16—C17—Fe1—C1	−75.20 (18)
Fe1—C15—C16—C17	58.55 (17)	C18—C17—Fe1—C1	166.02 (13)
C14—C15—C16—Fe1	−58.62 (17)	C16—C17—Fe1—C15	37.09 (15)
C15—C16—C17—C18	0.2 (3)	C18—C17—Fe1—C15	−81.70 (15)
Fe1—C16—C17—C18	59.10 (16)	C18—C17—Fe1—C16	−118.8 (2)
C15—C16—C17—Fe1	−58.86 (18)	C16—C17—Fe1—C3	−159.67 (15)
C15—C14—C18—C17	0.3 (3)	C18—C17—Fe1—C3	81.54 (16)
Fe1—C14—C18—C17	−59.85 (16)	C16—C17—Fe1—C2	−117.27 (16)
C15—C14—C18—C19	−179.0 (2)	C18—C17—Fe1—C2	123.94 (15)
Fe1—C14—C18—C19	120.9 (3)	C2—C1—Fe1—C4	81.09 (16)
C15—C14—C18—Fe1	60.12 (17)	C5—C1—Fe1—C4	−38.19 (15)
C16—C17—C18—C14	−0.3 (3)	C2—C1—Fe1—C5	119.3 (2)
Fe1—C17—C18—C14	59.74 (16)	C2—C1—Fe1—C18	−45.2 (4)
C16—C17—C18—C19	179.0 (2)	C5—C1—Fe1—C18	−164.5 (3)
Fe1—C17—C18—C19	−121.0 (2)	C2—C1—Fe1—C14	165.4 (2)
C16—C17—C18—Fe1	−60.05 (16)	C5—C1—Fe1—C14	46.1 (3)
C14—C18—C19—O3	176.3 (2)	C2—C1—Fe1—C17	−77.55 (18)
C17—C18—C19—O3	−2.8 (4)	C5—C1—Fe1—C17	163.17 (14)
Fe1—C18—C19—O3	−91.9 (3)	C2—C1—Fe1—C15	−160.85 (15)
C14—C18—C19—O4	−2.1 (4)	C5—C1—Fe1—C15	79.87 (17)
C17—C18—C19—O4	178.8 (2)	C2—C1—Fe1—C16	−118.56 (16)
Fe1—C18—C19—O4	89.7 (2)	C5—C1—Fe1—C16	122.15 (15)
O4—C20—C21—C26	−41.8 (3)	C2—C1—Fe1—C3	37.32 (15)
O4—C20—C21—C22	139.6 (2)	C5—C1—Fe1—C3	−81.97 (15)
C26—C21—C22—C23	−1.4 (4)	C5—C1—Fe1—C2	−119.3 (2)
C20—C21—C22—C23	177.3 (3)	C14—C15—Fe1—C4	−74.74 (17)
C21—C22—C23—C24	2.0 (4)	C16—C15—Fe1—C4	165.68 (15)
C22—C23—C24—C25	−1.3 (4)	C14—C15—Fe1—C5	−116.27 (15)
C23—C24—C25—C26	0.2 (4)	C16—C15—Fe1—C5	124.14 (15)
C24—C25—C26—C21	0.3 (4)	C14—C15—Fe1—C18	38.18 (14)
C22—C21—C26—C25	0.3 (4)	C16—C15—Fe1—C18	−81.40 (16)
C20—C21—C26—C25	−178.4 (3)	C16—C15—Fe1—C14	−119.6 (2)
O1—C6—O2—C7	5.8 (4)	C14—C15—Fe1—C17	82.55 (16)
C5—C6—O2—C7	−173.27 (19)	C16—C15—Fe1—C17	−37.03 (15)
C8—C7—O2—C6	86.9 (3)	C14—C15—Fe1—C1	−158.54 (14)
O3—C19—O4—C20	−1.9 (4)	C16—C15—Fe1—C1	81.88 (17)
C18—C19—O4—C20	176.5 (2)	C14—C15—Fe1—C16	119.6 (2)
C21—C20—O4—C19	114.0 (2)	C14—C15—Fe1—C3	−46.2 (4)
C3—C4—Fe1—C5	−118.3 (2)	C16—C15—Fe1—C3	−165.8 (3)
C3—C4—Fe1—C18	83.51 (17)	C14—C15—Fe1—C2	170.1 (2)
C5—C4—Fe1—C18	−158.14 (14)	C16—C15—Fe1—C2	50.6 (3)
C3—C4—Fe1—C14	125.93 (15)	C17—C16—Fe1—C4	−159.6 (3)
C5—C4—Fe1—C14	−115.72 (14)	C15—C16—Fe1—C4	−39.4 (4)
C3—C4—Fe1—C17	51.1 (3)	C17—C16—Fe1—C5	166.02 (14)
C5—C4—Fe1—C17	169.4 (2)	C15—C16—Fe1—C5	−73.83 (18)
C3—C4—Fe1—C1	−80.39 (16)	C17—C16—Fe1—C18	−38.32 (14)
C5—C4—Fe1—C1	37.96 (14)	C15—C16—Fe1—C18	81.83 (16)
C3—C4—Fe1—C15	166.73 (15)	C17—C16—Fe1—C14	−82.68 (15)
C5—C4—Fe1—C15	−74.92 (17)	C15—C16—Fe1—C14	37.47 (15)
C3—C4—Fe1—C16	−163.5 (3)	C15—C16—Fe1—C17	120.1 (2)
C5—C4—Fe1—C16	−45.2 (4)	C17—C16—Fe1—C1	123.86 (15)
C5—C4—Fe1—C3	118.3 (2)	C15—C16—Fe1—C1	−115.99 (15)
C3—C4—Fe1—C2	−36.78 (15)	C17—C16—Fe1—C15	−120.1 (2)
C5—C4—Fe1—C2	81.57 (16)	C17—C16—Fe1—C3	48.5 (3)
C1—C5—Fe1—C4	118.9 (2)	C15—C16—Fe1—C3	168.7 (2)
C6—C5—Fe1—C4	−123.2 (3)	C17—C16—Fe1—C2	81.71 (17)
C4—C5—Fe1—C18	50.1 (3)	C15—C16—Fe1—C2	−158.14 (15)
C1—C5—Fe1—C18	169.1 (2)	C2—C3—Fe1—C4	−120.5 (2)
C6—C5—Fe1—C18	−73.1 (3)	C4—C3—Fe1—C5	38.61 (14)
C4—C5—Fe1—C14	81.72 (16)	C2—C3—Fe1—C5	−81.84 (16)
C1—C5—Fe1—C14	−159.36 (14)	C4—C3—Fe1—C18	−115.19 (16)
C6—C5—Fe1—C14	−41.5 (2)	C2—C3—Fe1—C18	124.36 (16)
C4—C5—Fe1—C17	−166.8 (3)	C4—C3—Fe1—C14	−72.65 (18)
C1—C5—Fe1—C17	−47.9 (4)	C2—C3—Fe1—C14	166.89 (15)
C6—C5—Fe1—C17	70.0 (4)	C4—C3—Fe1—C17	−158.53 (15)
C4—C5—Fe1—C1	−118.9 (2)	C2—C3—Fe1—C17	81.02 (17)
C6—C5—Fe1—C1	117.9 (3)	C4—C3—Fe1—C1	82.91 (16)
C4—C5—Fe1—C15	123.56 (15)	C2—C3—Fe1—C1	−37.55 (15)
C1—C5—Fe1—C15	−117.51 (15)	C4—C3—Fe1—C15	−37.5 (4)
C6—C5—Fe1—C15	0.4 (2)	C2—C3—Fe1—C15	−157.9 (3)
C4—C5—Fe1—C16	163.94 (15)	C4—C3—Fe1—C16	166.5 (2)
C1—C5—Fe1—C16	−77.14 (17)	C2—C3—Fe1—C16	46.0 (3)
C6—C5—Fe1—C16	40.7 (2)	C4—C3—Fe1—C2	120.5 (2)
C4—C5—Fe1—C3	−38.01 (15)	C3—C2—Fe1—C4	37.09 (15)
C1—C5—Fe1—C3	80.91 (15)	C1—C2—Fe1—C4	−82.43 (16)
C6—C5—Fe1—C3	−161.2 (2)	C3—C2—Fe1—C5	81.59 (16)
C4—C5—Fe1—C2	−81.43 (16)	C1—C2—Fe1—C5	−37.93 (14)
C1—C5—Fe1—C2	37.50 (14)	C3—C2—Fe1—C18	−75.60 (18)
C6—C5—Fe1—C2	155.4 (2)	C1—C2—Fe1—C18	164.88 (15)
C14—C18—Fe1—C4	80.06 (16)	C3—C2—Fe1—C14	−38.9 (4)
C17—C18—Fe1—C4	−161.05 (15)	C1—C2—Fe1—C14	−158.4 (3)
C19—C18—Fe1—C4	−42.9 (3)	C3—C2—Fe1—C17	−118.08 (16)
C14—C18—Fe1—C5	44.1 (3)	C1—C2—Fe1—C17	122.40 (15)
C17—C18—Fe1—C5	163.0 (2)	C3—C2—Fe1—C1	119.5 (2)
C19—C18—Fe1—C5	−78.8 (3)	C3—C2—Fe1—C15	163.3 (2)
C17—C18—Fe1—C14	118.9 (2)	C1—C2—Fe1—C15	43.8 (3)
C19—C18—Fe1—C14	−123.0 (3)	C3—C2—Fe1—C16	−160.62 (14)
C14—C18—Fe1—C17	−118.9 (2)	C1—C2—Fe1—C16	79.86 (18)
C19—C18—Fe1—C17	118.2 (3)	C1—C2—Fe1—C3	−119.5 (2)

### Hydrogen-bond geometry (Å, °)

**Table:**

*D*—H···*A*	*D*—H	H···*A*	*D*···*A*	*D*—H···*A*
C1—H1···O2^i^	0.95	2.46	3.368 (3)	160
C7—H7B···O1^ii^	0.99	2.44	3.350 (3)	152
C9—H9···O1^ii^	0.95	2.59	3.374 (3)	141
C20—H20A···O3	0.99	2.28	2.716 (3)	106
C20—H20B···O3^ii^	0.99	2.54	3.421 (3)	148
C22—H22···O3^ii^	0.95	2.51	3.233 (3)	133

Symmetry codes: (i) *x*, *y*−1, *z*; (ii) *x*, *y*+1, *z*.
